# The effect of teaching on awareness and attitude of the students of Zanjan Branch, Islamic Azad University, about AIDS

**DOI:** 10.1186/1742-4690-9-S1-P126

**Published:** 2012-05-25

**Authors:** SeyedehSusan Raoufikelachayeh

**Affiliations:** 1 University Department of Nursing, Zanjan Branch, Islamic Azad University, Zanjan, Iran

## Introduction and objectives

AIDS is an acquired syndrome of immunity damage caused by body immunity damaging virus. As our population is young and consciousness raising has an essential role in prevention of a disease and also the young people are the main victims of it, so it is necessary to determine student’s knowledge and attitude as a symbol of young population to understand their ability in campaign against the AIDS and distinguish appropriate educational programs.

## Materials and methods

This semi experimental study was performed on 150 engineering students of Islamic Azad University of Zanjan Branch. Samples were chosen by random systematic method. The data collection tool was a questionnaire concluding three parts of demographic characteristics, knowledge level, and attitude questions. After performing the pre-test, the educational program was held during three weeks and then the post–test was held. The data was analyzed by SPSS software and the scores which were achieved by samples before and after the educational program were compared using t-test and paired t-test.

## Results

This study showed that 64/6 % were male and 36/4 were female. The knowledge rate of the students about risk factors of the disease before and after intervention were 16/03 2/04 and 18/02 1/44 respectively. The mean and standard deviation of attitude score of the students also increased from 90/16 10/6 to 96/60 10/33 after the education.

## Conclusions

Findings of this research show the positive effects of education on knowledge and attitude of the students, and providing education and appropriate background in educational environment seems necessary for employing effective behaviors. Keywords: AIDS, Attitude, Education, knowledge.

